# Neuron-specific transcriptomic signatures indicate neuroinflammation and altered neuronal activity in ASD temporal cortex

**DOI:** 10.1073/pnas.2206758120

**Published:** 2023-03-02

**Authors:** Pan Zhang, Alicja Omanska, Bradley P. Ander, Michael J. Gandal, Boryana Stamova, Cynthia M. Schumann

**Affiliations:** ^a^Department of Psychiatry and Biobehavioral Sciences, David Geffen School of Medicine, University of California, Los Angeles, CA 90095; ^b^Department of Human Genetics, David Geffen School of Medicine, University of California, Los Angeles, CA 90095; ^c^Semel Institute for Neuroscience and Human Behavior, University of California, Los Angeles, CA 90095; ^d^Department of Psychiatry and Behavioral Sciences, School of Medicine, University of California, Davis, Sacramento, CA 95817; ^e^University of California, Davis, MIND Institute, Sacramento, CA 95817; ^f^Department of Neurology, University of California, Davis, School of Medicine, Sacramento, CA 95817; ^g^Department of Psychiatry, Perelman School of Medicine, University of Pennsylvania, Philadelphia, PA 19104; ^h^Department of Genetics, Perelman School of Medicine, University of Pennsylvania, Philadelphia, PA 19104; ^i^Lifespan Brain Institute, Penn Med and the Children’s Hospital of Philadelphia, Philadelphia, PA 19104

**Keywords:** ASD, transcriptome, neuron-specific

## Abstract

Autism spectrum disorder (ASD) is a highly heterogeneous disorder, yet transcriptomic profiling of bulk brain tissue has identified substantial convergence among dysregulated genes and pathways in ASD. However, this approach lacks cell-specific resolution. We performed comprehensive transcriptomic analyses on bulk tissue and laser-capture microdissected (LCM) neurons from 59 postmortem human brains (27 ASD and 32 controls) in the superior temporal gyrus (STG) of individuals ranging from 2 to 73 years of age. In bulk tissue, synaptic signaling, heat shock protein-related pathways, and RNA splicing were significantly altered in ASD. There was age-dependent dysregulation of genes involved in gamma aminobutyric acid (GABA) (*GAD1* and *GAD2*) and glutamate (*SLC38A1*) signaling pathways. In LCM neurons, AP-1-mediated neuroinflammation and insulin/IGF-1 signaling pathways were upregulated in ASD, while mitochondrial function, ribosome, and spliceosome components were downregulated. GABA synthesizing enzymes *GAD1* and *GAD2* were both downregulated in ASD neurons. Mechanistic modeling suggested a direct link between inflammation and ASD in neurons, and prioritized inflammation-associated genes for future study. Alterations in small nucleolar RNAs (snoRNAs) associated with splicing events suggested interplay between snoRNA dysregulation and splicing disruption in neurons of individuals with ASD. Our findings supported the fundamental hypothesis of altered neuronal communication in ASD, demonstrated that inflammation was elevated at least in part in ASD neurons, and may reveal windows of opportunity for biotherapeutics to target the trajectory of gene expression and clinical manifestation of ASD throughout the human lifespan.

## Significance

We present a comprehensive assessment of neuronal cell-type-specific gene expression and alternative splicing changes in ASD cortex, directly comparing RNA-seq results from bulk tissue with isolated neurons. We observe strong signatures of cell stress and neural-immune/inflammatory pathway activation present within ASD neurons—a signal that is typically attributed to astrocyte/microglial populations. Our findings also provide further evidence for the hypothesized imbalance of excitatory to inhibitory neuronal activity in the brains of individuals with ASD. Moreover, we find that the transcriptomic architecture of ASD interacts substantially with age, thus revealing windows of opportunity for treatments that target specific molecular pathology.

Autism spectrum disorder (ASD) defines a heterogeneous set of complex neurodevelopmental disorders affecting 1 in 54 children in the USA according to current estimation (1, 2) and confers lifelong challenges. ASD is characterized by difficulties with social communication as well as a repetitive, restricted repertoire of behaviors and interests (3). Population, family, and twin studies all indicate a strong genetic component contributing to risk for ASDs (4, 5), with heritability estimates of ~70% (6). However, the genetic causes and pathophysiology of ASD are varied and often complex.

Despite this heterogeneity, transcriptomic analyses of postmortem human brain have elucidated substantial convergent molecular-level pathology associated with idiopathic and syndromic forms of ASD (7–13). Multiple studies have profiled the transcriptomes of postmortem brain regions from individuals diagnosed with ASD (7, 8, 11, 13), including the temporal cortex implicated due to its critical importance in speech and language function (7, 8, 11, 13). The most consistent findings include disruption of neuronal/synaptic activity and activation of innate immunity/glial markers (7, 8, 11). Alternative splicing and non-coding RNAs have also been shown to be dysregulated in ASD brains (8).

Most previous transcriptomic studies, however, profiled homogenate brain tissue and have therefore been unable to pinpoint the underlying specific cell types in which gene expression is altered. Single-nucleus RNA-sequencing (sn-RNAseq) datasets have started to be generate in postmortem ASD cortex (12, 13), identifying substantial changes in upper-layer excitatory neurons and microglia, consistent with observations from bulk tissue. As such sn-RNAseq datasets currently primarily profile the 3′ end of highly expressed genes within each cell, these data characterize neither lowly expressed coding and noncoding genes, nor splicing alterations that may contribute to altered neuronal function in ASD.

**Fig. 1.**
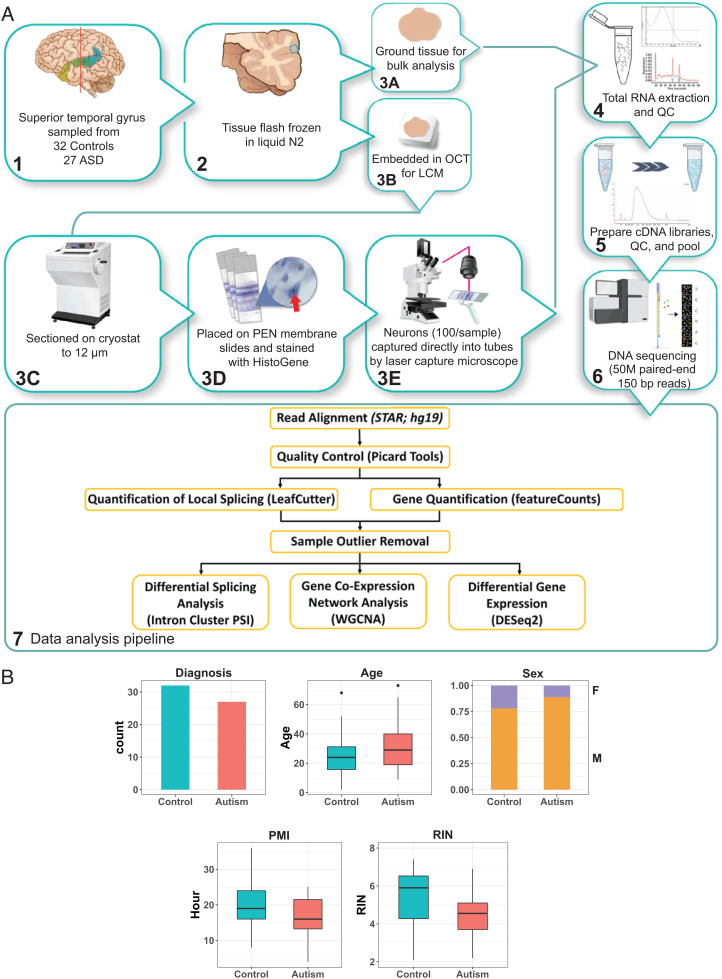
Overview of Experiment Design, Data Analysis Pipeline (*A*), and Study Cohort (*B*).

Here, we performed the systematic study using transcriptomic profiling to directly compare both bulk cortical tissue and laser capture microdissected (LCM) neurons from anatomically well-defined superior temporal gyrus (STG) samples from 59 subjects (27 with ASD and 32 controls) ranging from 2 to 73 years of age (Fig. 1 and Dataset S12). The STG modulates language processing and social perception, thereby playing a critical role in integrating a breadth of information to provide meaning to the surrounding world (14). Structural and functional imaging studies have long implicated STG in ASD (14, 15); however, molecular-level changes in neurons remain unknown. This study aimed to identify neuron-specific transcriptomic changes in ASD brain by identifying differentially expressed genes, differential splicing (DS) events, age-related gene expression changes across the lifespan, and co-expression networks to reveal gene modules altered in ASD. We also applied mechanistic modeling approaches to pinpoint pathways and genes directly linked to ASD in neurons. (Fig. 1).

## Results

### Global Gene Expression Changes in ASD STG.

**Fig. 2.**
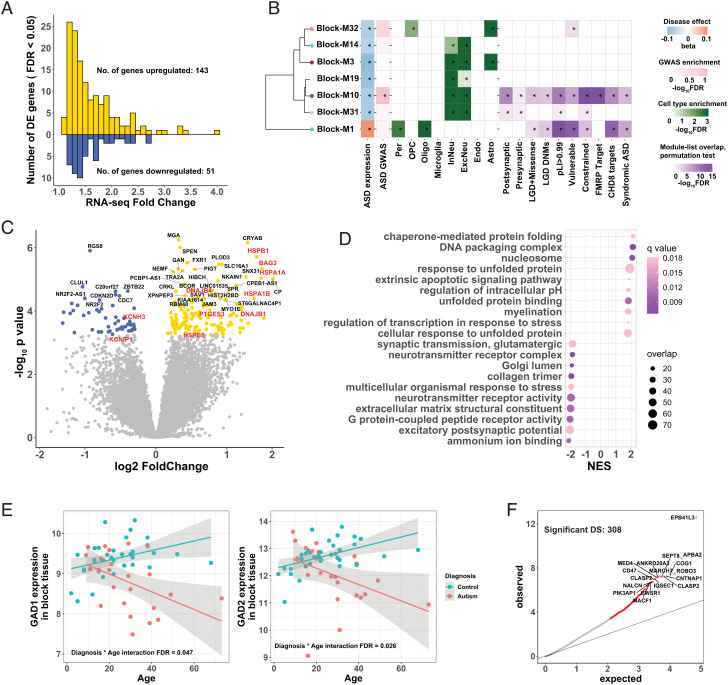
Transcriptomic Difference Between ASD Cases and Controls in Bulk Tissue STG. (*A*) Distribution of fold-change of differential expression for 194 differentially expressed genes. Case/control fold-changes for upregulated genes are plotted in gold (N = 143, positive values) and control/case fold-changes for downregulated genes in blue (N = 51, negative values). (*B*) Co-expressed gene modules that were significantly disrupted in ASD. Modules were hierarchically clustered by module eigengene. Module-diagnosis associations were shown on the right of each module (*FDR < 0.05). Additional enrichment analyses were also shown for each module, including: Enrichment for ASD GWAS common variants (33)(*FDR < 0.1); Enrichment for major CNS cell types (16)(*FDR < 0.05); Enrichment against literature-curated gene lists (*FDR < 0.05, see *SI Appendix*, *SI Methods* for details of the gene lists). Per, pericytes; OPC, oligodendrocyte progenitor cells; InNeu, inhibitory neuron; ExcNeu, excitatory neuron; Oligo, oligodendrocytes; Endo, endothelial cells; Astro, astrocytes. (*C*) Volcano plot showing significantly up- (gold) and down-regulated (blue) genes (FDR < 0.05). Genes discussed in the main text are colored red. (*D*) Functional enrichment of differentially expressed genes in ASD cases compared to controls. Top 10 significantly enriched up- and down-regulated categories were shown. The color of each dot reflects FDR-corrected q-value, and the size of each dot reflects the number of overlapped genes between our gene list and the corresponding GO category. NES, normalized enrichment score. (*E*) Age trajectory of GAD1 (*Left*) and GAD2 (*Right*) gene expression, stratified by ASD diagnosis. The expression levels were on the log2CPM (counts per million) scale. (*F*) Quantile-quantile plot of observed *P*-values vs. expected *P*-values for differentially spliced intron clusters. Significant DS events (FDR < 0.05) were colored red, and overlapping gene names were labeled for top clusters.

RNA sequencing was performed on bulk tissue STG of 59 human brains, 27 from individuals with ASD and 32 from neurotypical controls, ranging from 2 to 73 years of age. Following quality control, we comprehensively characterized differential gene expression (DGE) and local splicing alterations in ASD. After adjusting for known covariates and correcting for multiple comparisons, we found 194 differentially expressed genes between individuals with ASD and controls (FDR < 0.05). Of these, 143 were upregulated and 51 were downregulated (Fig. 2*A* and Dataset S1), with a median absolute fold change of 1.45 (range 1.11 to 4.04, Fig. 2*A*). We observed significant concordance between our DGE results and previous data of the same region from different samples (13) (*SI Appendix*, Fig. S1, Spearman ρ = 0.37 for t statistics among all genes, *P* value < 10^−16^).

Functional and pathway enrichment analyses indicated an over-representation of heat-shock proteins (HSPs) and HSP-related chaperones, which were upregulated in ASD subjects. This included HSP70 family members *HSPA1A* and *HSPA1B*; HSP40 family members *DNAJB1* and *DNAJB4*; small HSP20 family members *HSPB1* and *HSPB8*; and HSP-binding chaperons *BAG3* and *PTGES3* (Fig. 2 *C* and *D*). HSPs are involved in stress response, immune activation, and inflammation(17, 18), all of which were upregulated in ASD postmortem brain (7). Downregulated genes were mainly enriched in pathways related to synaptic function (Fig. 2*D*), consistent with previous findings (7). Notably, two important voltage-gated potassium channel-related genes *KCNH3* and *KCNIP1* were among the most downregulated (Fig. 2*C*), which may relate to disrupted neuronal excitability hypothesized in ASD (19, 20).

As age-dependent expression alterations have been reported in ASD brain (21), we employed an analytical model accounting for age and the interaction between age and diagnosis. Fourteen genes showed age-dependent DGE between ASD and control (Dataset S2). Gene set enrichment analysis (GSEA) indicated that genes with significant diagnosis-by-age interaction were enriched in immune/inflammation pathways and synaptic-related pathways. (*SI Appendix*, Fig. S9).

Interestingly, genes involved in gamma aminobutyric acid (GABA) synthesis (*GAD1* and *GAD2*) (22) were downregulated in ASD only during late adulthood (Fig. 2*E*). This may indicate an age-dependent dysregulation of GABA signaling in ASD neurons, or a decrease in the proportion of GABAergic neurons in ASD brains (23).

Given mounting evidence suggesting potential mechanistic overlap between neurodevelopmental and neurodegenerative disorders, particularly with respect to genes involved in synapse and brain connectivity (24), we evaluated the overlap between our ASD findings with published results in Alzheimer’s disease (AD). Using results from Mount Sinai/JJ Peters VA Medical Center Brain Bank (MSBB) cohort (25), we demonstrated that the changes we detected in ASD were significantly overlapped with transcriptomic alterations observed in AD from the same brain region (Odds Ratio = 1.6, *P* = 1.9 × 10^−8^, *SI Appendix*, Fig. S8).

DS events in the bulk tissue transcriptome were evaluated using LeafCutter (26). Among 35,505 intron clusters identified by LeafCutter, 308 clusters (297 unique genes) showed significant DS between ASD patients and controls (FDR < 0.05). The 297 genes did not show significant functional enrichment (Fig. 2*F* and Dataset S5).

To place subtle changes across the ASD STG transcriptome into a systems-level context, we performed weighted gene correlation network analysis (WGCNA) to build gene co-expression networks (27), identifying 31 modules of co-expressed genes (*Methods* and Dataset S3). Seven modules significantly associated with ASD diagnosis, two of which were strongly enriched for ASD-associated genetic risk factors (Modules Block-M1 and Block-M10, Fig. 2*B*).

Module Block-M1 was upregulated in ASD STG and its gene members were enriched in RNA splicing and mRNA metabolic pathways (Dataset S4). Notably, significantly upregulated HSPs were also members of the Block-M1 module (Dataset S3). HSPs contribute to RNA splicing during stress (28). Downregulated modules in ASD were mostly enriched for synaptic functions (Block-M3, Block-M10, Block-M14, Block-M19, Block-M31; Dataset S4). Cell-type enrichment analysis also indicated these downregulated modules were enriched in marker genes for both excitatory and inhibitory neurons (Fig. 2*B*), suggesting a broad disruption of neuronal and synaptic processes in ASD STG.

Genes in the upregulated module Block-M1 and one downregulated module (Block-M10) were enriched in high-confidence ASD risk loci (29, 30), mutationally constrained (31), and highly intolerant to mutations (pLI > 0.99) genes (32), as well as regulatory target genes of CHD8, which has clear links to at least a subset of ASD cases (33) (Fig. 2*B*, see *SI Appendix*, *SI Methods* for the details of all curated, hypothesis-driven gene sets. Dataset S14). Many hub genes for module Block-M10 encoded synaptic proteins (*SI Appendix*, Fig. S2 and Dataset S3). This module was also enriched for ASD common risk alleles from ASD GWAS data (34). Together this suggested a causal role of synaptic dysfunction in ASD etiology.

### Neuron-Specific Gene Expression and Splicing Alterations in ASD STG.

**Fig. 3.**
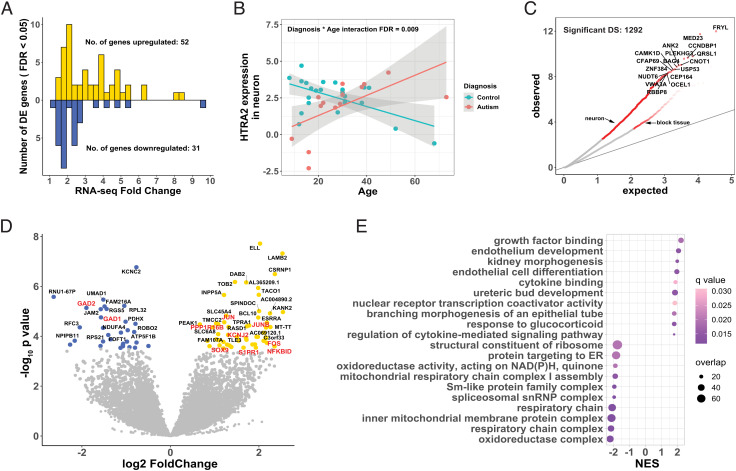
DGE and DS Between ASD Cases and Controls in LCM Neurons from STG. (*A*) Distribution of fold-change of differential expression for 83 differentially expressed genes. Case/control fold-changes for upregulated genes are plotted in gold (N = 52, positive values) and control/case fold-changes for downregulated genes in blue (N = 31, negative values). (*B*) Age trajectory of HTRA2 gene expression, stratified by ASD diagnosis. The expression levels are on the log_2_CPM (counts per million) scale. (*C*) Quantile–quantile plot of observed *P*-values vs. expected *P*-values for differentially spliced intron clusters. Significant DS events (FDR < 0.05) were colored red, and overlapping gene names were labeled for top clusters. DS results from bulk tissue were also plotted for comparison. (*D*) Volcano plot showing up- (gold) and down-regulated (blue) genes. Genes discussed in the main text are colored red. (*E*) Functional enrichment of differentially expressed genes in ASD cases compared to controls. Top 10 significantly enriched up- and downregulated categories are shown. The color of each dot reflects FDR-corrected q-value, and the size of each dot reflects the number of overlapped genes between our gene list and the corresponding GO category. NES, normalized enrichment score**.**

To provide cell-type specificity for the observed transcriptomic changes, we performed laser capture microdissection to capture neurons using STG sections taken from the same subjects profiled using bulk RNA-seq (*SI Appendix*, Figs. S6 and S7). We then interrogated ASD-associated gene expression and splicing alterations using the same bioinformatic pipelines as above. Across 13,458 neuron-expressed genes, 83 were significantly differentially expressed between ASD subjects and controls at FDR <0.05, of which 52 were upregulated and 31 downregulated (Fig. 3*A* and Dataset S6). Median absolute fold change in expression between subjects with ASD and controls was 2.48 (range 1.29 to 9.72; Fig. 3*A*). The concordance of neuronal DGE with bulk tissue DGE was low (Spearman ρ = 0.18 for t statistics across all genes, *SI Appendix*, Fig. S3), suggesting our analysis captured ASD signatures unique to neurons.

Upregulated genes in ASD neurons were highly enriched in pathways related to growth and differentiation (Fig. 3*E*). Specifically, the AP-1 transcription factor complex components (35) *FOS*, *JUN* and *JUNB* were all upregulated in ASD neurons, among other growth/differentiation regulators such as *SOX9*, *S1PR1*, and *PPP1R16B* (Fig. 3*D*). The AP-1 transcription factor complex is known to regulate many downstream biological processes (35). Indeed, we found upregulated B cell signaling adaptor gene *BCL10* and NFκB inhibitor delta gene *NFKBID* in ASD neurons (Fig. 3*D*). Up-regulation of both *NFKBID* and AP-1 point to dysregulated inflammation in ASD neurons. In addition, the inward-rectifier potassium ion channel gene *KCNJ2* was also upregulated in ASD neurons (Fig. 3*D*). Interestingly, both *KCNJ2* and AP-1 subunit *FOS* were involved in regulating excitability and plasticity at the cholinergic synapse (36–38).

Downregulated genes in ASD neurons are primarily enriched in mitochondrial function and oxidoreductase activity (Fig. 3*E*). Specifically, comparing to bulk tissue STG, more subunits of the NADH:ubiquinone oxidoreductase (complex I) were downregulated in neurons, and their effect sizes were larger (*SI Appendix*, Fig. S4). Our results provide evidence that compared to bulk tissue, mitochondrial dysfunction is much more profound in STG neurons.

While LCM captured both excitatory and inhibitory neurons, we note that *GAD1* and *GAD2* genes are among the most downregulated in ASD neurons (Fig. 3*D*). The coordinated down-regulation of both GABA synthesizing enzymes suggest that the level of GABA neurotransmitter may be decreased in ASD neurons, providing support to the excitation to inhibition (E/I) imbalance hypothesis of ASD (19, 20, 39).

By testing the interaction between diagnosis and age, three genes (*HTRA2,* aka *OMI*; *ZNF765*; and *PCDHB18P*) showed age-dependent differential expression in ASD neurons (Dataset S7; no pathway enrichment was detected for neuronal genes with significant diagnosis-by-age interaction). For example, age trajectories of serine peptidase *HTRA2* were opposite in ASD brains compared to controls (Fig. 3*B*). In healthy brains, the expression of *HTRA2* was much higher before age 30 and decreases with age, while its expression levels begin lower and increase with age in ASD STG neurons.

To further evaluate potential overlap between ASD and neurodegenerative processes in neurons, we compared our neuronal findings with results from MSBB. Changes in ASD neuronal transcriptome were also significantly overlapped with transcriptomic alterations observed in AD from the same brain region (odds ratio = 1.4, *P* = 0.01, *SI Appendix*, Fig. S8).

We also quantified local splicing events in the neuronal transcriptome. After adjusting for multiple testing, LeafCutter identified 1,292 significant differential spliced intron clusters (1,177 unique genes) out of 17,250 total intron clusters at FDR < 0.05 (Fig. 3*C* and Dataset S8). No functional enrichment was observed for the 1,177 genes. We observed more disruptions in local splicing events in ASD neurons than in bulk tissue (308 DS out of 35,505 events in bulk tissue, 1,292 DS out of 17,250 events in neurons; *P* < 2 × 10^−16^ test of proportions).

### Neuron-Specific Networks Pinpoint Subtle Changes in the Neuronal Transcriptome in ASD.

**Fig. 4.**
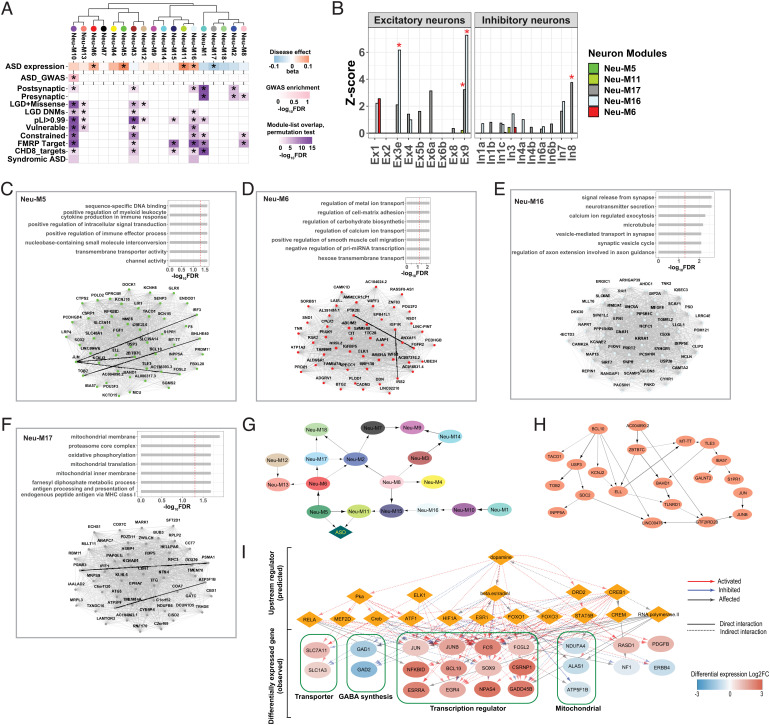
Co-expression and Mechanistic Network Analysis of the ASD Neuronal Transcriptome. (*A*) Hierarchical clustering of neuronal gene co-expression modules by module eigengenes. Module–diagnosis associations were shown below each module. Enrichment for ASD GWAS common variants is shown for each module. Enrichment against literature-curated gene lists is shown on the bottom. (*B*) Module enrichment for neuron subtypes. Expression profiles of neuron subtypes were obtained from ref. 16. Red asterisks indicate significant enrichment. (*C*–*F*) Functional enrichment (*Top*) and top 50 hub genes (*Bottom*) for module Neu-M5 (*C*), Neu-M6 (*D*), Neu-M16 (*E*) and Neu-M17 (*F*). Edges represent co-expression (Pearson correlation > 0.5). Co-expressed partners with evidence of protein-protein interaction (PPI) were connected by solid black lines. PPI data was compiled from well-characterized PPI databases (*SI Appendix*, *SI Methods*). (*G*) Bayesian network representing the relationships among modules (ellipse) and ASD diagnosis (diamond). The thickness of the arrow is proportional to the number of times that a connection was detected during a bootstrap of 500 times. (*H*) The estimated gene regulatory network (Bayesian network) for 21 hub genes in Neu-M5. (*I*) Dopamine network built by IPA from neuron DEGs (FDR < 0.1, 225 genes). A network of 17 upstream regulators (orange diamonds) were predicted to drive the changes of 74 observed target genes (23 of which were shown based on node connectivity ≥ 2, ellipse). ELK1 was differentially expressed in neurons, and it was also predicted as an upstream regulator.

Co-expression network analysis on neuronal data identified 18 modules, each containing between 101 and 998 co-expressed genes (Dataset S9). Four modules were significantly upregulated in ASD neurons, while one module was downregulated (Fig. 4*A*).

The upregulated neu-M5 co-expression module was highly represented by the DGE analysis signal. Upregulated genes *JUN, JUNB, and NFKBID* were all hub genes of neu-M5 module. Neu-M5 module also captured additional AP-1 subunits and interactors, such as *FOSL2* (40) and *IRF3* (41, 42). Neu-M5 module was enriched in immune response pathways, providing further evidence that AP-1-mediated neuroinflammation was elevated in ASD neurons (Fig. 4*C* and Dataset S10). Hubs of Neu-M5 also contained multiple ion channel-related genes, such as sodium ion channel gene *SCN1B*, potassium channel genes *KCNJ2* and *KCNJ10*, and solute carrier gene *SLC40A1* (Fig. 4*C* and Dataset S9). Coordinated upregulation of various ion channels suggested that membrane transport was activated in ASD neurons, consistent with heightened excitability. Neu-M5 was significantly overlapped with module M16 from Voineagu et al. (7)*.* M16 was also enriched in immune/inflammatory response and was up-regulated in ASD (7). Our data refined our understanding of the neuroinflammatory changes in ASD to include a neuronal component. Additionally, downregulated neu-M17 module was enriched in mitochondrial function and contained the most differentially expressed mitochondrial genes, such as ATP synthase subunits *ATP5F1B* and *ATP5PF* (Fig. 4*F* and Dataset S9).

Neuronal co-expression networks further captured signals that were not detected by DGE analysis. Neu-M6 module was upregulated in ASD, and among its hub genes were several insulin signaling pathway components, including insulin-like growth factor (IGF) receptor *IGF1R*, IGF binding protein *IGFBP5*, insulin receptor substrate *IRS2* as well as CBL-associated *SORBS1* (Fig. 4*D* and Dataset S9). Our results provided direct molecular-level evidence that insulin signaling was altered in ASD neurons.

Among all five significantly disrupted modules, none were enriched for ASD common variants and only one upregulated module (Neu-M16) showed enrichment in highly confident ASD risk genes, as well as in several other curated gene sets (Fig. 4*A*). Neu-M16 was enriched in synaptic functions (Fig. 4*E* and Dataset S10). Further, cell-type analysis showed that Neu-M16 was also highly enriched in excitatory neurons (Fig. 4*B*), with *CAMK2A* and *CAMK2B* among its hub genes (Dataset S9). The upregulation of Neu-M16 suggested elevated excitatory signal in ASD neurons.

We further tested whether significantly disrupted neuronal modules were enriched in any neuron subtypes. Upregulated neuronal modules in ASD were only enriched in excitatory neuron subtypes, while enrichment of inhibitory neurons was only observed for downregulated modules (Fig. 4*B*). This provides additional evidence for altered neuronal activity in ASD neurons, consistent with the findings in our DGE analysis.

To gain mechanistic insights from our neuronal data, we used Bayesian network inference (43–45) to distinguish between direct module-trait associations and indirect module-trait correlations. Our Bayesian network contained 19 nodes, including 18 neuronal co-expressed gene modules represented by module eigengenes, plus a binary node representing ASD diagnosis. Our results indicated that Neu-M5 was directly associated with ASD, conditioned on all other modules (Fig. 4*G*). This provided further evidence that neuroinflammation was directly associated with ASD in neurons.

We further used Bayesian network to prioritize among hub genes of Neu-M5 module for future validation. We focused on 21 genes within Neu-M5 that were also significant differentially expressed genes (DEGs). The Bayesian network learned from these 21 hub genes is shown in Fig. 4*H*. Genes with the highest degree of connectivity in this network included *BCL10, ELL_,_* and *GTF2IRD2B*, which can be potential targets for functional evaluations.

As an orthogonal approach, we identified the upstream regulators and built mechanistic networks in Ingenuity Pathway Analysis (IPA® ) with the differentially expressed genes in captured neurons (FDR < 0.1, 225 genes, Dataset S13). Dopamine was predicted as a top upstream regulator with the mechanistic network presented in Fig. 4*I*. This dopamine network contained 17 upstream regulators predicted to drive the observed changes of 74 target genes, including AP-1 subunits *JUN, JUNB, FOS*, and GABA synthetase *GAD1* and *GAD2*. Moreover, there were seven genes (*BCL10, JUN, JUNB, KCNJ2, ELL, S1PR1,* and *SDC2*) from this mechanistic network that overlapped with the 21 genes identified by the Bayesian network analysis. This further highlighted the involvement of these genes in ASD, since they were identified independently by different modeling approaches.

### Small Noncoding RNAs Are Selectively Downregulated in ASD Neurons and Correlate with Altered Local Splicing.

**Fig. 5.**
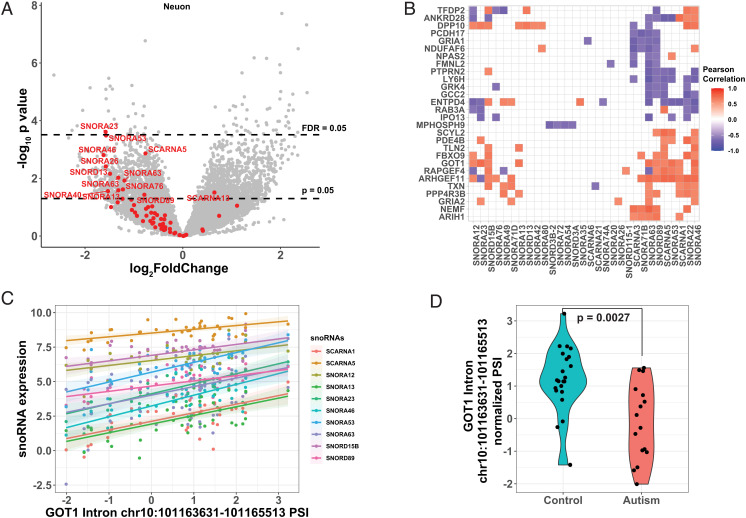
Coordinated Dysregulation of snoRNAs in ASD Neurons. (*A*) Volcano plot showing differentially expressed genes in ASD neurons compared to control. snoRNA genes were colored red. (*B*) Significant correlation between snoRNA expression (x-axis) and intron PSI (y-axis). Introns are labeled with the name of overlapping gene locus. Gene loci that are correlated with more than 3 snoRNAs were shown. (*C*) Scatter plot showing the correlation between GOT1 intron 5 PSI and expression levels of multiple snoRNAs across all neuron samples. Also shown were fitted regression lines with 95% CIs. Intron coordinates were based on GRCh37. (*D*), PSI of GOT1 intron 5 is downregulated in ASD neurons.

When investigating the genes downregulated in ASD neurons more closely, we noticed a striking pattern that 51 out of the 59 neuron-expressed small nucleolar RNA (snoRNA) (46) genes were downregulated in ASD neurons, and 13 were significantly downregulated at *P*-value < 0.05 (Fig. 5*A* and Dataset S6). Dysregulation of snoRNAs was not observed in bulk tissue (Dataset S1), and snoRNAs were undetectable in a recent ASD single-cell study (12). snoRNAs are involved in the modification and maturation of ribosomal RNAs (rRNAs) and small nuclear RNAs (snRNAs) (47). Interestingly, both ribosome and spliceosome components were among the most downregulated in ASD neurons (Fig. 3*E*). Moreover, snRNAs were also downregulated in ASD neurons, with 23 out of 24 snRNA genes downregulated in ASD and 13 significantly downregulated at P value < 0.05 (*SI Appendix*, Fig. S5 and Dataset S6) Table 1.

**Table 1.** Summary of genes discussed in this manuscript

Noted Categories	Genes
E/I signal	CAMK2A^*^; CAMK2B^*^; GAD1^*^; GAD2^*^; GOT1^*^
Growth / differentiation	FOS^*^; FOSL2^*^; IRF3^*^; JUN^*^; JUNB^*^; PPP1R16B^*^; S1PR1^*^; SOX9^*^
Heat shock	*BAG3^^†^^; DNAJB1^^†^^; DNAJB4^^†^^; HSPA1A^^†^^; HSPA1B^^†^^; HSPB1^^†^^; HSPB8^^†^^; PTGES3^^†^^*
Immune / inflammation	BCL10^*^; NFKBID^*^
Insulin signaling	IGF1R^*^; IGFBP5^*^; IRS2^*^; SORBS1^*^
Ion channel	KCNH3*^†^*; KCNIP1^^†^^; KCNJ10^*^; KCNJ2^*^; SCN1B^*^; SLC40A1^*^
Mitochondrial	ATP5F1B^*^; ATP5PF^*^
Age-associated	GAD1^^†^^; GAD2^^†^^; HTRA2^*^; PCDHB18P^*^; ZNF765^*^

^*^observed in STG neurons.

^†^observed in bulk STG.

The category of each gene is based on what we discussed in this manuscript.

As snoRNAs and snRNAs are known to be critical regulators of alternative splicing (48–51), and splicing alterations are strongly implicated in ASD and observed in LCM-captured neurons, we next examined alterations in splicing events that may be associated with snoRNA dysregulation. We calculated the correlation between snoRNA expression level and each local splicing event in neuronal data, followed by FDR correction for multiple comparisons. We identified 835 gene loci in neurons with at least one intron whose percent spliced in (PSI) was significantly correlated with snoRNA gene expression (Dataset S11). Of these 835 intron clusters, 196 were significantly dysregulated in ASD neurons (Dataset S11). Several intron clusters correlated with multiple snoRNAs and corresponded to genes involved in synaptic functions (Fig. 5*B*). For example, *GOT1* encodes the glutamic-oxaloacetic transaminase known to function as an essential regulator of glutamate level (52). PSI of intron 5 of *GOT1* was highly correlated with the expression level of multiple snoRNA genes (Fig. 5*C*). *GOT1* intron 5 was also differentially spliced between ASD and control (Fig. 5*D*). DS of *GOT1* gene may change the level of glutamate and thus leads to an imbalance of E/I in neuronal communication in ASD neurons.

## Discussion

### Bulk Tissue Transcriptome Findings Reveal Downregulated Neuronal and Synaptic Function Processes, Upregulation of HSPs, and Unfolded Protein Response (UPR) in ASD Temporal Cortex.

Our bulk tissue analyses revealed a potential causal role of downregulated neuronal processes and synaptic functions in ASD etiology, consistent with findings from previous bulk-tissue transcriptomic studies on the same brain region (7). Previous studies also reported dysregulated alternative splicing events in ASD brain (8, 53). DS analysis in bulk STG found several synaptic genes, including *CANCA2D1*, *CAMK4*, *CLASP2*, *CNTNAP1*, *EPHB1*, *KALRN*, *NRXN3*, *SOS2* and *SYNGAP1*, differentially spliced in ASD. *SYNGAP1* isoforms have been shown to differentially regulate synaptic plasticity and dendritic development (54). This further signifies the importance of studying alternatively spliced isoforms in ASD brain.

We also observed a coordinated upregulation of multiple HSPs and HSP-related chaperones in ASD STG. HSPs can serve as activators and regulators of the immune system (18), and upregulated HSPs may induce immune responses in ASD brain. They also play a role in facilitating alternative RNA splicing (28). Previous studies found that both immune response and RNA splicing are upregulated in ASD brain (7, 8, 11), and our results signify that upregulated HSP-related pathways potentially contribute to these observations. HSPs and HSP-related chaperones are normally induced in response to stress. The upregulation of HSPs in ASD neurons may relate to elevated endoplasmic reticulum (ER) stress since both UPR and apoptosis are also upregulated in our ASD bulk data (Fig. 2*D*). ASD-linked rare or de novo mutations in synaptic genes can lead to misfolded proteins and cause ER stress (55), itself coupled to heightened inflammation and neurotoxic cell death (56). ER stress-related genes are also dysregulated in the middle frontal cortex of subjects with ASD (57). Our data suggest that ER stress is a major response to ASD genetic mutations, and ER stress activates UPR, including the production of HSPs and chaperones. UPR further induces multiple downstream processes such as inflammation and immune response (58). Limiting the effect of ER stress and UPR may be a promising therapeutic avenue for ASD.

### ASD Neuronal Transcriptome Reveals Upregulated Neuroinflammation and Altered Neuronal Activity.

We observed a strong upregulation of AP-1 transcription factor components in ASD neurons. AP-1 subunits *FOS*, *JUN* and *JUNB* were upregulated at FDR < 0.05, and *FOSL2* was upregulated at nominal *P* < 0.05. AP-1 regulated gene expression in response to various stimuli, including cytokines, growth factors, stress signals, infections and inflammation/neuroinflammation (35). In ASD neurons, AP-1 activation likely induces broad inflammatory response, since several immune and inflammation-related genes were also strongly upregulated. These included *NFKBID* and *BCL10*, which were involved in the NF-κB pathway and were upregulated at FDR < 0.05. In addition, the interferon regulatory factor *IRF3* is upregulated at *P* < 0.05, and *IRF3* is co-expressed with AP-1 subunits. The simultaneous upregulation of AP-1 subunits, NFκB-related genes and interferon regulatory factors suggested that immune and inflammation responses were activated in ASD neurons. Upregulated immune response and neuroinflammation have been consistently observed in ASD patients by bulk tissue transcriptomic studies largely implicating glial cells (7, 8). However, our results demonstrated that immune/neuroinflammatory response was clearly activated in ASD neurons, and may be mediated by transcription factor AP-1.

We also observed strong downregulation in ASD STG neurons of *GAD1* and *GAD2* genes, involved in the biosynthesis of the inhibitory neurotransmitter GABA. In contrast, *CAMK2A* and *CAMK2B* genes, which are essential for aspects of plasticity at glutamatergic excitatory synapses, are upregulated at nominal significance. In addition, co-expressed gene modules that were upregulated in ASD neurons were mainly enriched in excitatory neurons, while the downregulated module was primarily enriched in inhibitory neurons. These data supported the hypothesis that ASD reflects imbalance of E/I in neuronal communication, also reported in several brain regions in ASD (19, 20, 39). This is a report providing molecular-level evidence for imbalance of E/I in neuronal communication specifically in STG neurons in ASD.

Future studies will focus on the role of snoRNAs in ASD neurons, and other long and small modulatory non-coding RNAs. Given the emerging role of snoRNAs as alternative splicing regulators (50, 51), we hypothesize that a coordinated downregulation of multiple snoRNAs correlates with elevated dysregulation of local splicing events in ASD neurons. Our data provide evidence supporting this hypothesis; however, no causal relationship can be determined. It will be critical to determine if snoRNA dysregulation plays a causal role in ASD etiology, and if so, pinpoint the underlying mechanism and possibly relate to transcript isoforms driving ASD brain and neuronal phenotype.

In our study, the concordance of ASD-associated transcriptomic changes in bulk tissue and LCM neurons was relatively low. This is not surprising given bulk tissue contains various different neuronal and non-neuronal cell types. Neuron-specific changes could be masked by changes in other cell types when profiling bulk tissue. The lack of concordance highlighted the necessity of specifically isolating and profiling each cell type to identify cell-type-specific transcriptomic signatures.

### Age-Associated Differential Expression in STG Points to Altered Neuronal Activity in ASD.

In bulk tissue, genes involved in GABAergic signaling (*GAD1* and *GAD2*) were upregulated with age in controls, while downregulated with age in ASD. Multiple lines of evidence have pointed to deviations in the production, transmission, and reception of GABAergic inhibitory interneurons, including decreased numbers of GABAergic interneurons (especially Parvalbumin neurons) (59) and reduced density of GABA receptors (60–62). Our findings indicated that the reduction of *GAD1* and *GAD2* mRNA levels in ASD brain became more profound with age, consistent with the observations at cellular level. In addition, *SLC38A1*, involved in neurotransmission at glutaminergic and GABAergic synapses (63), was downregulated in ASD relative to control STG. *SLC38A1* is implicated in Rett Syndrome (64) and mitochondrial disorders, and its decrease may contribute to the observed alterations in synapse formation and neural connectivity.

In LCM neurons, the expression of *HTRA2* was higher below age 30 and decreases with age in control neurons, while lower at younger ages and increasing with age in ASD neurons. HTRA2 is important in maintaining mitochondrial homeostasis (65) and inducing apoptosis. It is implicated in pathogenesis of neurodegeneration, hypoxic-ischemic damage, and is proposed as a potential treatment target in neurological diseases (66). Attenuated *HTRA2* activity may lead to neuronal cell death, altered chaperon activity and autophagy and has been linked to Parkinson’s disease (67). In addition, increased active form of the *OMI/HTRA2* serine protease has been positively correlated with cholinergic alterations in AD brain (68). Thus, it is plausible that the altered expression of *HTRA2* with age we observed in ASD brain may be associated with neuronal alterations during development. These findings further support the hypothesis of altered neuronal E/I activity, neuroinflammation, cell death, and mitochondrial dysfunction, implicated in ASD (69) and suggest treatment windows to target specific genes to alter their expression trajectories with age.

In summary, our age-related findings support the premise brain development in individuals with ASD deviates from that of the neurotypical trajectory beginning in childhood and continues to evolve across the lifespan (70, 71). Although the STG remains relatively unexplored (72–74), other brain regions display early excess followed by reductions in volume, connectivity, and cell densities as people with ASD age through adulthood. Initial excess and overconnectivity may lead to hyperexcitation, rendering the brain vulnerable to age-related and pro-inflammatory mechanisms contributing to later degenerative outcomes. Our findings of altered neuroinflammatory expression patterns, taken together with reports of excessive microglial activation in STG (74, 75), implicate immune dysfunction in the pathophysiology of ASD that may exacerbate with age. Additionally, we found significant relationships with our ASD transcriptome profiles and AD profiles in the same brain region (25), supporting recent theories of increased susceptibility to neurodegenerative and cognitive decline (76, 77). Lastly, our findings of an age-related decrease in *GAD* expression in bulk STG tissue, and an overall downregulation of *GAD* in neurons, further supports the hypothesis that GABAergic inhibitory neurons are disproportionally affected in ASD throughout the lifespan.

### Mechanistic Modeling Highlighted Pathways and Genes That Were Directly Associated with ASD in Neurons.

We applied two orthogonal approaches to gain mechanistic insight from our LCM neuron data. Bayesian network inference built a network from co-expressed modules and further prioritized hub genes within selected module(s), identifying *BCL10, ELL* and *GTF2IRD2B* as genes having driving roles in ASD. The approach from Ingenuity Pathways Analyses first identified upstream regulators that align with the observed expression changes in our dataset and elucidated that they share a predicted association with dopamine signaling pathways. Expansion of the regulatory network to include another layer of DEGs in our dataset indicated strong overlap and regulatory connections to many key genes involved in neuronal function and inflammation (*GAD1, GAD2,* and many transcriptional regulators). Interestingly, the expansion of the IPA network further converged on regulatory molecules *BCL10* and RNA polymerase II, of which the former was directly identified in the independent Bayesian approach, and the latter associated with *ELL*. Together these two independent approaches highlight the role of inflammatory pathways and genes in ASD etiology.

## Limitation and Future Studies

Although our study had a relatively large sample size of 59 human brains, there are inherent limitations to utilizing postmortem human brain tissue, including variability in cause of death, co-morbid conditions, medication use, postmortem interval (PMI), etc., Future studies will have the advantage of more in-depth clinical information available from newer brain tissue collections such as Autism BrainNet (SFARI) and the NIH Biobanks to evaluate the relationship of cellular and molecular findings with clinical characteristics. Most of our subjects were male, consistent with the 4:1 ratio of males/females with ASD; future studies should include a larger female cohort to adequately evaluate sex differences. Additionally, the development of novel tools for profiling the transcriptome such as scRNA-seq allows for analyses of individual cell types at high resolution; however, snRNA-seq currently does not provide the depth of coverage to quantify low-expressed genes and local splicing, especially in human brain samples that we were able to achieve with LCM neuron samples. Our study, however, did not distinguish neuronal subtypes, as we found Histogene the most reliable cell body staining method for maintaining tissue quality and accurate neuronal identification. Our future studies will expand on current findings, particularly age-related changes in *GAD* implicating GABAergic interneurons, to map expression in specific cell types in multiple brain regions, in relation to clinical characteristics, across the human lifespan.

## Methods

### Bulk Tissue RNA Extraction and Library Preparation.

Tissue and clinical data collection procedures were approved by the institutional review board (IRB) and Human and Anatomical Specimens Tissue Oversight Committee (HASTOC) at the University of California, Davis School of Medicine. Informed consent was obtained from next-of-kin at the time of brain tissue collection for follow-up to collect donor clinical information to confirm diagnoses by the Autism Tissue Program (now Autism BrainNet).

Human brain tissue was collected, sectioned coronally and flash-frozen. The STG was identified anatomically according to “Atlas of the Human Brain” fourth edition (Maj, Majtanik, Paxinos 2015). Total RNA was extracted using the Direct-zol RNA MiniPrep (Zymo Research #R2051).

Fifty nanograms of RNA from each sample were used to create strand-specific total RNA libraries with the NuGEN Ovation Universal RNA-Seq System v2 and processed in parallel on the Sciclone NGS automated workstation (Perkin Elmer) according to manufacturer protocol.

### Laser Capture Microdissection, RNA Extraction, and Library Preparation.

Using a Leica LMD-6000 laser capture microdissection system, 100 neurons from each sample were laser captured directly into lysis buffer. RNA was extracted using the PicoPure total RNA kit according to manufacturer protocol. Strand-specific rRNA depleted RNA libraries were prepared from 10 μL of the final neuronal RNA.

### RNA Sequencing, Read Mapping, Quantification of Gene Expression, and QC.

RNA-Seq was performed on Illumina HiSeq4000. Libraries were sequenced to ~50 million 2 × 150 bp reads per sample. Reads were aligned to the GRCH37.p13 (hg19) reference genome using STAR. Gene-level quantifications were calculated using featureCounts (v1.6.4). Sample outliers were defined as samples with standardized sample network connectivity Z scores < −2 (78), and were removed. Using this method, five samples from bulk data, and five samples from neuron data were removed. Batch effects were accounted for using metrics derived from PicardTools (v2.21.2).

### DGE and Differential Alternative Splicing.

DGE analyses were performed using DESeq2 (1.22.2)(79) with default parameters. Local splicing analysis was performed using LeafCutter (26) as previously described (10).

### Co-expression Network Analysis.

Weighted gene co-expression network analysis (WGCNA) (27) was performed to define modules of co-expressed genes from RNA-seq data.

### Functional Enrichment Analysis.

Gene ontology (GO) enrichment was performed using the gProfileR R package (80) and the fGSEA algorithm as implemented in the clusterProfiler R package (81).

Enrichment analyses were also performed using several established, hypothesis-driven gene sets. Significance was determined from permutation-derived null distribution.

### Cell Type Enrichment Analysis.

Cell-type enrichment analysis was performed using the Expression Weighted Cell Type Enrichment (EWCE) package in R (82).

### GWAS Enrichment Analysis.

Stratified LD score regression (sLDSC) (83) was used to test whether a gene set of interest is enriched for SNP-heritability in a given GWAS dataset.

### Ingenuity Pathway Analysis.

Ingenuity Pathway Analysis (IPA®, QIAGEN) tools were used to predict the upstream regulators and to build mechanistic networks (84).

### Designing and Learning the Bayesian Network Structure.

Each of the 18 module eigengenes from our neuron co-expression data serves as one random variable (node) in our Bayesian network (BN). In addition, we also include diagnosis (ASD or CTL) as a binary random variable (node). This design allows us to distinguish between modules that are directly associated with ASD and those that are indirectly associated with ASD (45).

### Prioritizing Genes within Neu-M5.

To prioritize genes within the Neu-M5 module, we first identified 21 genes within Neu-M5 that are also significant DEGs. We then built Bayesian network among the 21 genes with method described in the above section. We selected genes with the highest number of connections as nodes likely to influence the expression of many other genes within the module.

### Alzheimer’s Disease Datasets.

Alzheimer's disease DGE data was obtained from the Mount Sinai/JJ Peters VA MSBB study (25) through Synapse via accession number syn30821563.

## Supplementary Material

Appendix 01 (PDF)

Dataset S01 (XLSX)

Dataset S02 (XLSX)

Dataset S03 (XLSX)

Dataset S04 (XLSX)

Dataset S05 (XLSX)

Dataset S06 (XLSX)

Dataset S07 (XLSX)

Dataset S08 (XLSX)

Dataset S09 (XLSX)

Dataset S010 (XLSX)

Dataset S011 (XLSX)

Dataset S012 (XLSX)

Dataset S013 (XLSX)

Dataset S014 (XLSX)

## Data Availability

All custom code used in this manuscript is available at https://github.com/gandallab/ASD_STG_LCM_RNAseq (85). RNA-seq data from this study is available through the database for Genotypes and Phenotypes (dbGaP Study Accession ID: phs003208) (86).
