# Supplementary material for: Neuron-specific transcriptomic signatures indicate neuroinflammation and altered neuronal activity in ASD temporal cortex
Source: Proc Natl Acad Sci U S A. 2023 Mar 2;120(10):e2206758120. doi: 10.1073/pnas.2206758120 (PMC10013873; doi:10.1073/pnas.2206758120)
Supplement: Supplementary file 1 — Appendix 01 (PDF) [file pnas.2206758120.sapp.pdf]

PNAS

Supporting Information for

Neuron-specific transcriptomic signatures indicate neuroinflammation and altered neuronal activity in ASD temporal cortex

Author list: Pan Zhang, Alicja Omanska, Bradley P. Ander, Michael J. Gandal, Boryana Stamova, Cynthia M. Schumann

Corresponding author: Cynthia M. Schumann (cschumann@ucdavis.edu); Boryana Stamova (bsstamova@ucdavis.edu); Michael J. Gandal (mgandal@mednet.ucla.edu).

This PDF file includes:

Figures S1 to S9
Legends for Datasets S1 to S14
SI Methods
SI References

Other supporting materials for this manuscript include the following:

Datasets S1 to S14

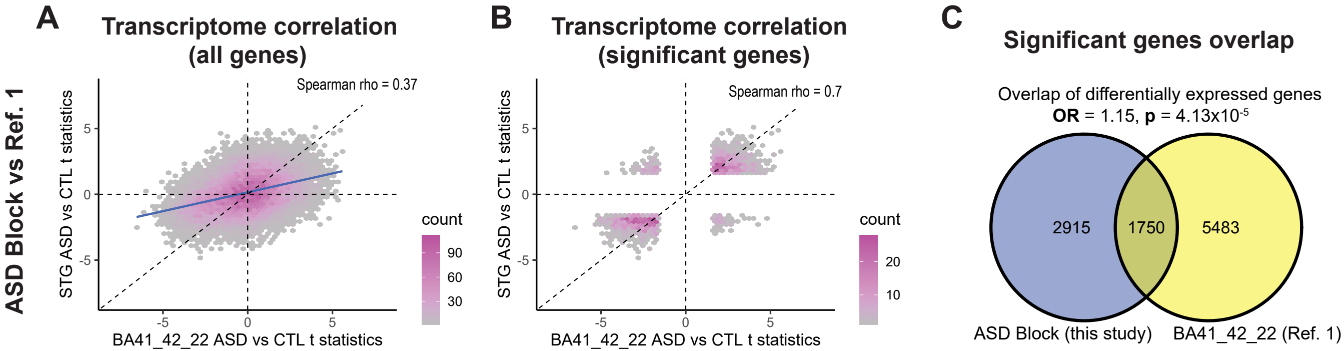

Supplementary Figure 1. Comparison of DEGs between bulk data from this study and that from a previous study (1). Binned density scatter plot comparing the t-statistics for all genes (A) and significant genes (nominal $p < 0.1$) (B) between this study (ASD vs control in bulk STG) and a previous study (ASD vs control in BA41, BA42, and BA22 bulk tissues). The overlap between significant DEGs were also tested by Fisher exact test with a total of 20677 genes as background.

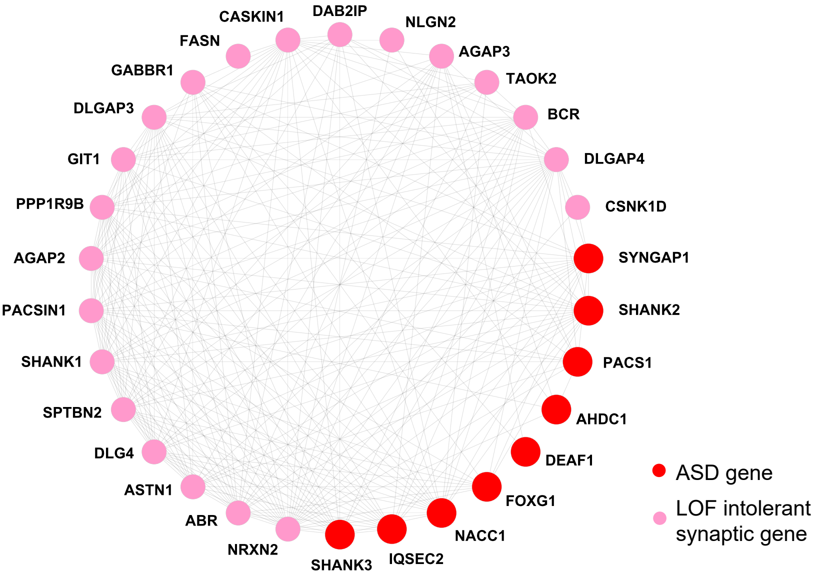

Supplementary Figure 2
Representative genes in module Block-M10.
Known ASD risk genes were colored red.
Synaptic genes that are intolerant to LOF mutation were colored pink.
Edges represent co-expression.

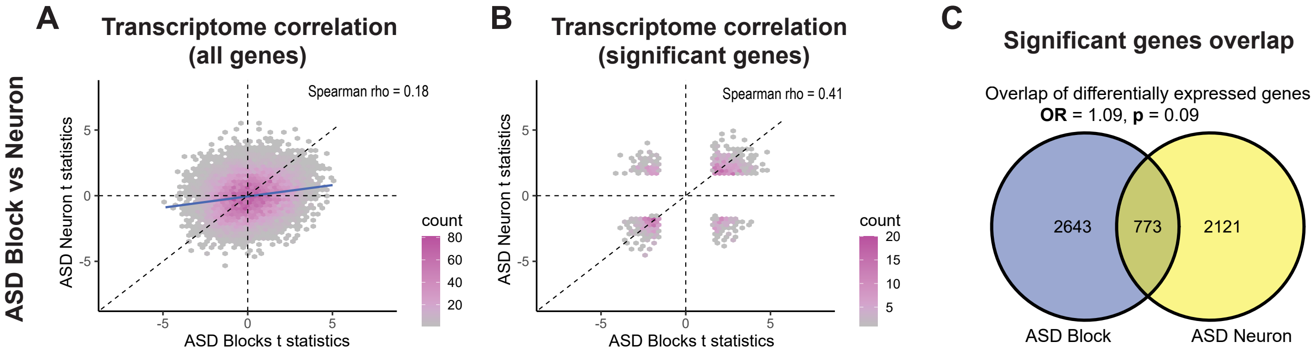

Supplementary Figure 3. Comparison of DEGs between bulk data and LCM neuron data. Binned density scatter plot comparing the t-statistics for all genes (A) and significant genes (nominal $p < 0.1$) (B) between neuron data and bulk tissue data. The overlap between significant DEGs were tested by Fisher's exact test with a total of 13411 genes as background.

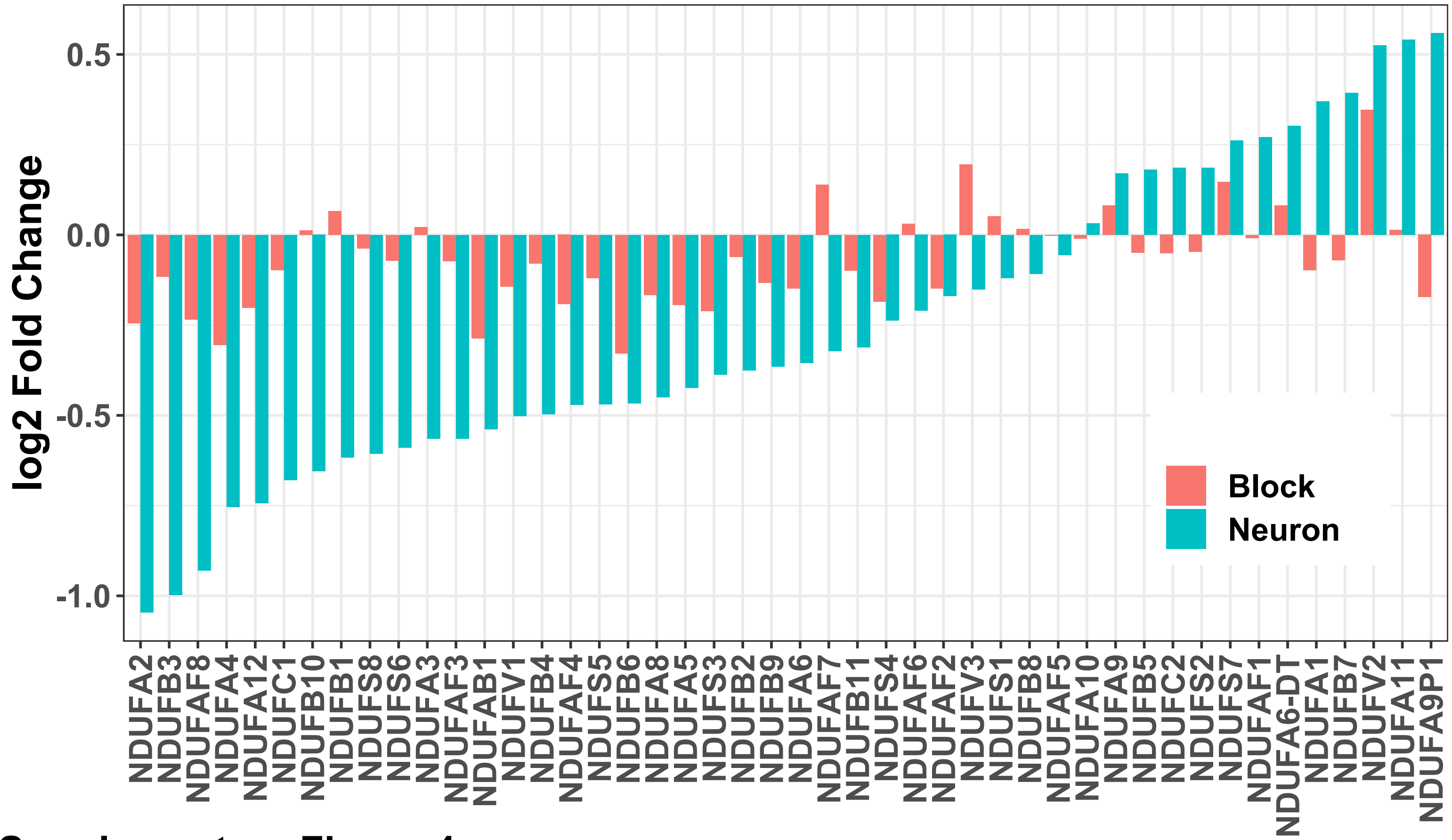

Supplementary Figure 4

Fold changes (ASD vs. CTL) of NADH:ubiquinone oxidoreductase (complex I) subunits in block tissue (red) and neurons (green).

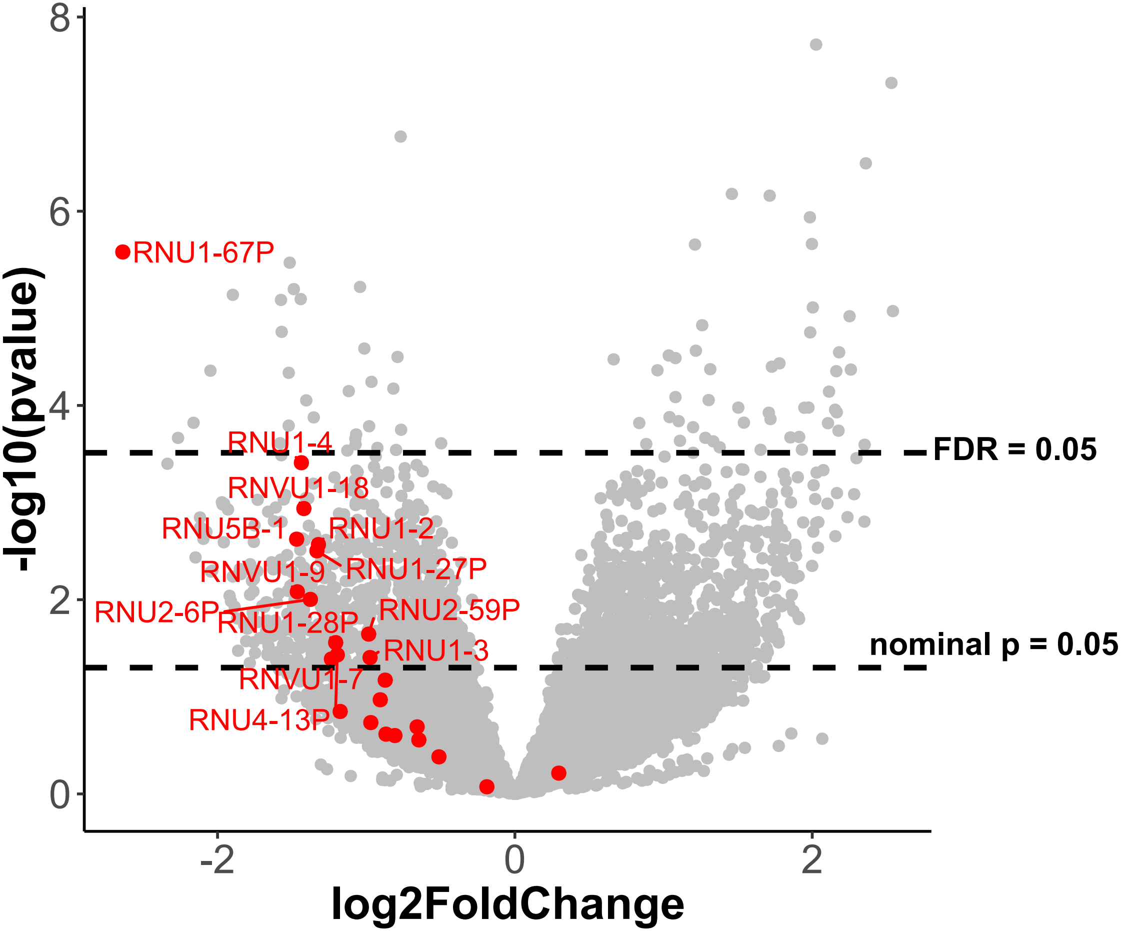

Supplementary Figure 5

Volcano plot showing differentially expressed genes in ASD neurons compared to control. snRNA genes were colored red.

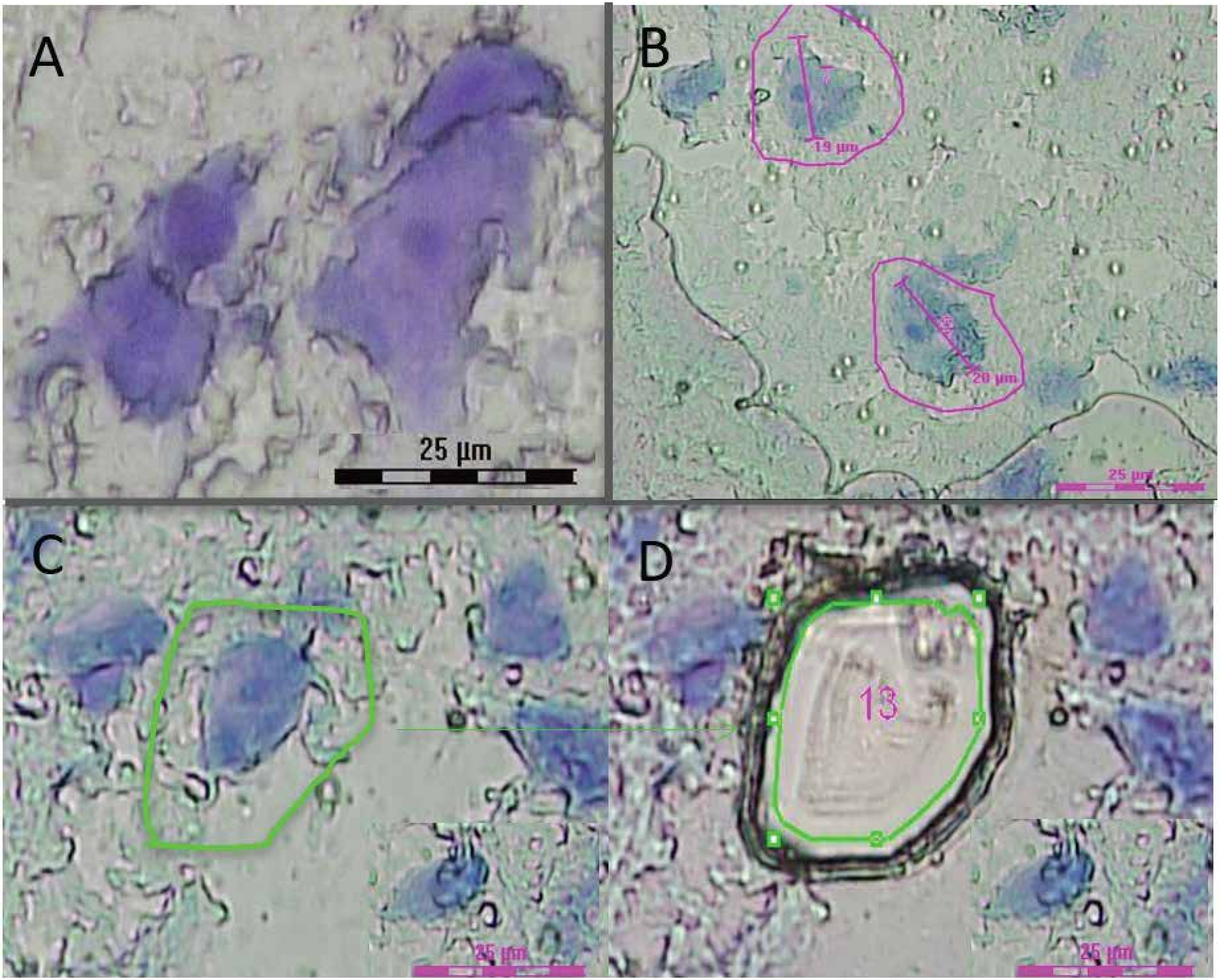

Supplementary Figure 6: Representative images of cell identification and capture during the laser capture microdissection protocol. Pyramidal neurons were stained by Histogene and identified by their distinct morphology (A). LCM targets were then selected for capture (B). The lower panels represent a neuronal target before (C) and after (D) successful capture.

Bulk Tissue final library BioAnalyzer

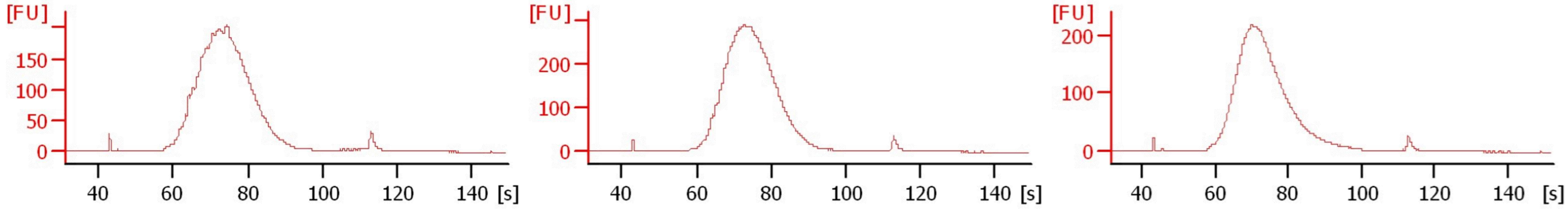

100 neuron LCM final library BioAnalyzer

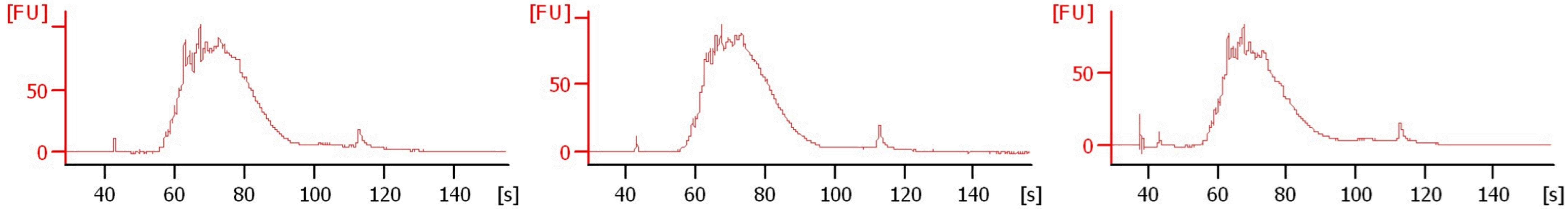

Pilot experiments of 25, 50 and 100 LCM neurons final library BioAnalyzer

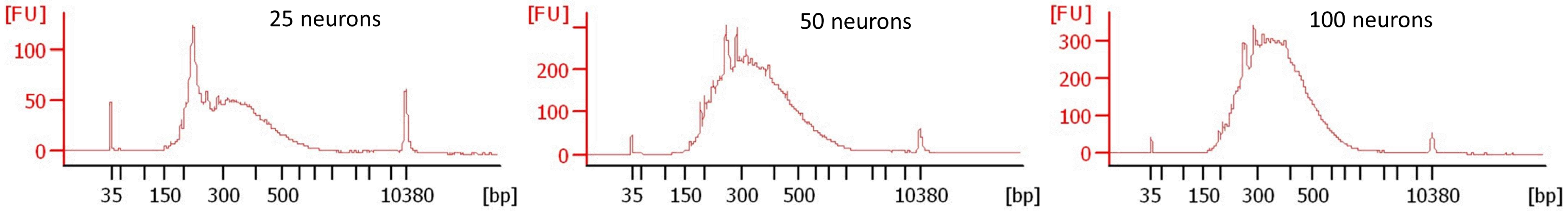

Supplementary Figure 7. Representative BioAnalyzer results of libraries prepared from LCM neurons and bulk brain tissue

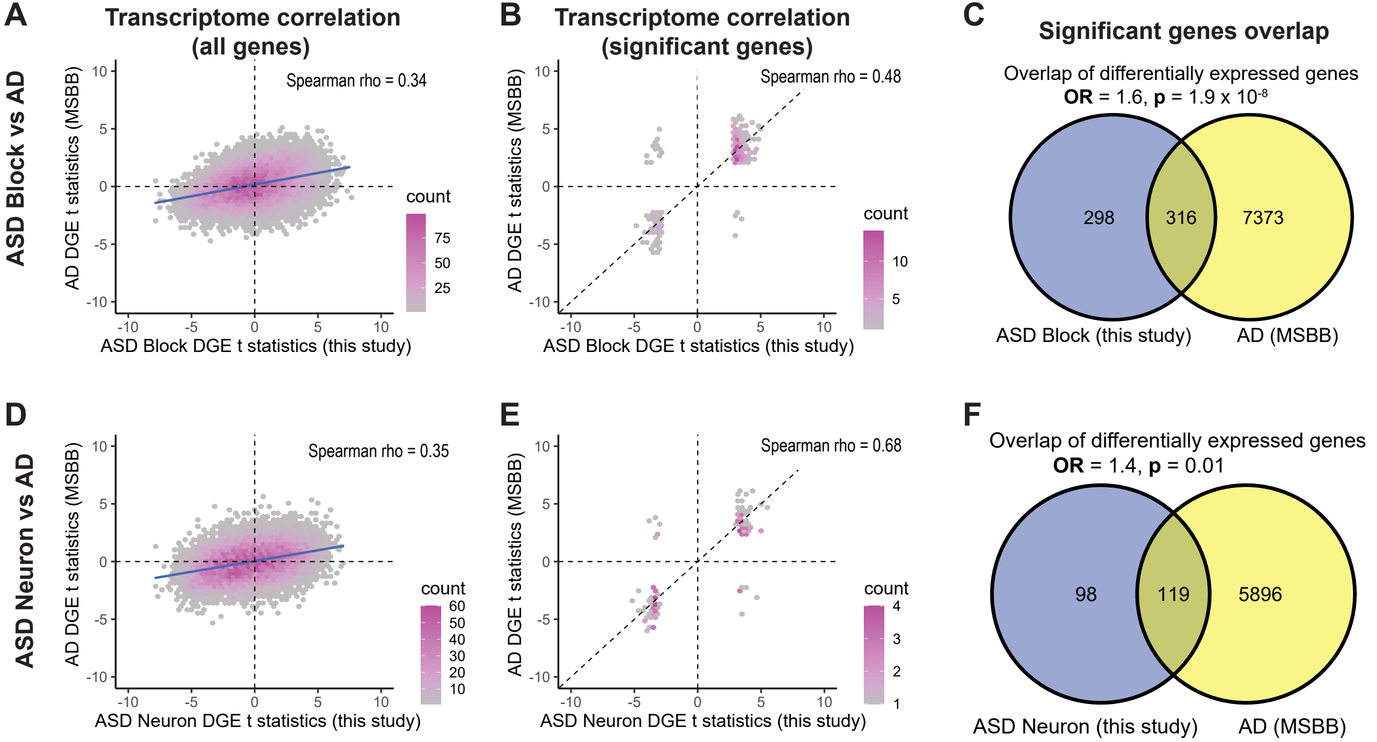

Supplementary Figure 8.

Transcriptome correlation and overlap of differentially expressed genes between ASD and AD

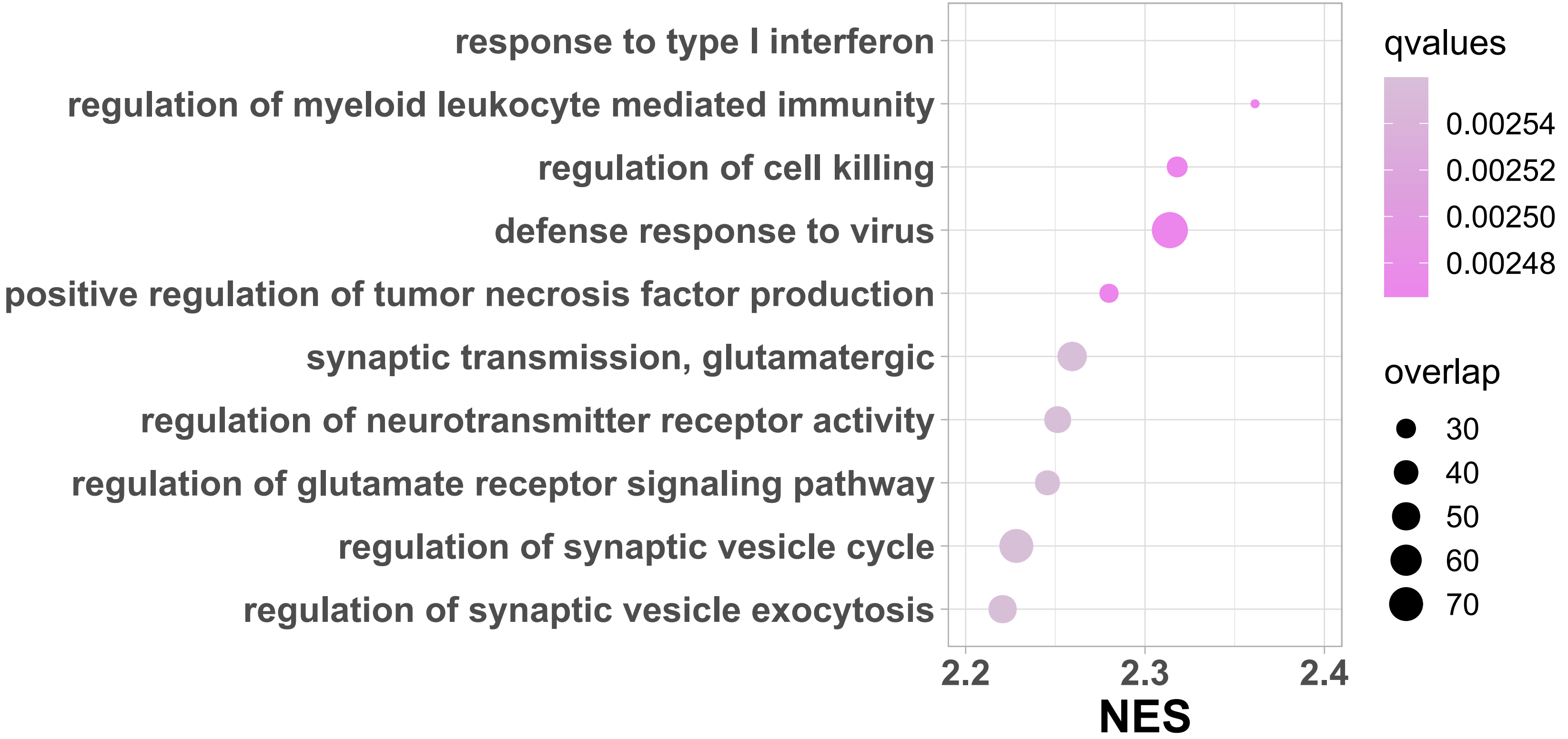

Supplementary Figure 9. Functional enrichment of genes with significant diagnosis-by-age interaction in block data. NES, normalized enrichment score from GSEA.

Dataset S1

DGE summary statistics for bulk tissue

Dataset S2

DGE summary statistics for age-diagnosis-interaction in bulk tissue

Dataset S3

Gene co-expression network module membership for bulk tissue

Dataset S4

Gene co-expression network module functional enrichment for bulk tissue

Dataset S5

DS summary statistics for bulk tissue

Dataset S6

DGE summary statistics for LCM neuron

Dataset S7

DGE summary statistics for age-diagnosis-interaction in LCM neuron

Dataset S8

DS summary statistics for LCM neuron

Dataset S9

Gene co-expression network module membership for LCM neuron

Dataset S10

Gene co-expression network module functional enrichment for LCM neuron

Dataset S11

Significant correlations between snoRNA gene expression and local splicing events

Dataset S12

Donor information

Dataset S13

Upstream regulator and mechanistic network analysis (from IPA®) of DEGs in ASD neuron

Dataset S14

Literature-derived, curated gene sets used for enrichment analysis

SI Methods

Bulk tissue RNA extraction and library preparation

Tissue and clinical data collection procedures were approved by the Institutional Review Board (IRB) and Human and Anatomical Specimens Tissue Oversight Committee (HASTOC) at the University of California, Davis School of Medicine. Informed consent was obtained from next-of-kin at the time of brain tissue collection for follow up to collect donor clinical information to confirm diagnoses by the Autism Tissue Program (now Autism BrainNet). Human brain tissue was collected, sectioned coronally and flash frozen. STG from 32 controls and 27 ASD cases (2-73 years old) was identified anatomically according to "Atlas of the Human Brain" 4th edition (Maj, Majtanik, Paxinos 2015). Brain tissue (18-25 mg) was excised from the STG and put directly into

600 µl of Tri Reagent lysis buffer. Total RNA was extracted using the Direct-zol RNA MiniPrep (Zymo Research #R2051) following manufacturer's protocol, with the inclusion of DNase I treatment and eluted in DNase/RNase-free water. Quality and quantity of RNA were determined via RNA 6000 Nano chip on 2100 Bioanalyzer (Agilent), NanoDrop 2000 spectrophotometer (ThermoFisher Scientific), and Qubit fluorometer (ThermoFisher Scientific).

From each of the STG samples, 50 ng of RNA were used to create strand-specific total RNA libraries with the NuGEN Ovation Universal RNA-Seq System v2 and processed in parallel on the Sciclone NGS automated workstation (Perkin Elmer) according to manufacturer protocol. Following second-strand cDNA synthesis, samples were sheared by sonication on the Covaris E220. InDA-C (aka AnyDeplete) primers were used to target and cleave adapters from rRNA transcripts before amplification of libraries through 16 cycles of PCR. Final barcoded libraries were bead purified and examined for QC using 2100 BioAnalyzer DNA High Sensitivity chips. Library concentration was calculated based on fragment size and normalized to 15 nM for sequencing.

Laser capture microdissection, RNA extraction and library preparation

Fresh-frozen STG tissue samples from 22 controls and 18 ASD cases (8-73 years old) were carefully dissected and embedded in OCT compound. The specimens were sectioned on a Microm HM550 cryostat (Thermo Scientific) at 12 µm and mounted on PEN membrane slides (ThermoFisher Scientific #LCM0522). Sections were hydrated with an ice-chilled ethanol series (100%, 75%, 50%) for 2 min each followed by HistoGene staining solution (ThermoFisher Scientific #KIT0415) for 30 seconds, 2 rinses in nuclease-free water, and alcohol dehydration (50%, 75%, 95%, 100% with molecular sieves). Slides were air dried and maintained on dry ice until laser capture microdissection.

Using a Leica LMD-6000 laser capture microdissection system, 100 neurons from each sample were laser captured directly into lysis buffer (PicoPure RNA Isolation Kit, ThermoFisher Scientific KIT0204). RNA was extracted using the PicoPure total RNA kit with inclusion of DNase I digest according to manufacturer protocol.

Strand-specific rRNA depleted RNA libraries were prepared from 10 µl of the final neuronal RNA eluate using the NuGEN Ovation SoLo Kit (NuGen #0500) for ultra-low input following manufacturer protocol with final amplification of 18 PCR cycles. Barcoded bead purified libraries were examined for QC using Qubit Fluorometer (ThermoFisher Scientific) and 2100 BioAnalyzer DNA High Sensitivity chips. Library concentration was calculated based on fragment size and normalized to 15 nM for sequencing.

RNA sequencing

Library concentrations were confirmed with qPCR and pooled before RNA-Seq was performed on Illumina HiSeq4000 at the Vincent J. Coates Genomics Sequencing Laboratory at the California Institute for Quantitative Biosciences (QB3) at University of California, Berkeley. Libraries from LCM samples and STG bulk were sequenced to about 50 million 2x150bp reads per sample. For libraries prepared with the NuGEN Ovation SoLo kit a Custom R1 primer (NuGEN) was used in place of the standard Illumina forward read primer.

Mapping, quantification of gene expression, and QC

RNA-seq reads were aligned to the GRCH37.p13 (hg19) reference genome via STAR (2.7.2a) using comprehensive gene annotations from GENCODE (v29 lifted over to hg19). Gene-level quantifications were calculated using featureCounts (v1.6.4), considering only uniquely-mapped reads. Quality control metrics were calculated using PicardTools (v2.21.2).

Gene-level counts were compiled and imported into R for downstream analyses. Expressed genes were defined as genes with non-zero count in at least 80% of samples. A total of 22,729 and 13,458 expressed genes from bulk tissue and LCM neurons, respectively, were used in the downstream analysis. Sample outliers were defined as samples with standardized sample network connectivity Z scores < -2 (2), and were removed. Using this method, 3 samples from bulk data, and 5 samples from neuron data were removed.

A set of 105 RNA-Seq quality control metrics from the outputs of PicardTools (CollectAlignmentSummaryMetrics, CollectInsertSizeMetrics, CollectRnaSeqMetrics, CollectGcBiasMetrics, MarkDuplicates) were compiled for each group of samples (bulk tissue and LCM neurons). These measures were summarized by the top principal components (termed seqPCs), which explained a significant portion of the total variance of each dataset. These seqPCs were used as potential covariates for downstream analysis.

Differential gene expression

Differential Gene Expression (DGE) analyses were performed using DESeq2 (1.22.2)(3) with default parameters. For bulk tissue data, diagnosis, sex, age, RNA integrity number (RIN), absorbance 260/280 ratio (A260/280) and top 3 seqPCs were used as covariates. For neuron data, diagnosis, sex, age, RNA library batch and top 3 seqPCs were used as covariates. To identify age-dependent differential expression, an interaction term between age and diagnosis was added to the above DESeq2 models.

Differential alternative splicing

Local splicing analysis was performed using LeafCutter (4) as previously described (5). In brief, Clusters of variable spliced introns across all samples were called first. Then differential splicing between ASD and control group was identified in each data set (bulk tissue and neuron) by jointly

modeling intron clusters using the Dirichlet-Multinomial generalized linear model (GLM). We controlled for the same covariates as above in the DGE analysis.

Intron clusters were first filtered to only keep clusters supported by at least 50 split reads across all samples, retaining introns of up to 100 kb and accounting for at least 1% of the total number of reads in the entire cluster. This intron count file was then used in the differential splicing (DS) analysis. For DS analysis, we further discarded introns that were not supported by at least one read in 5 or more samples. Clusters were then analyzed for DS if at least 3 samples in each comparison group (i.e. ASD or controls) had an overall coverage of 20 or more reads. P-values were corrected for multiple testing using the Benjamini-Hochberg (BH) method and used to select clusters with significant splicing differences (FDR < 0.1).

Co-expression network analysis

Weighted gene co-expression network analysis (WGCNA)(6) defined modules of co-expressed genes from RNA-seq data. All covariates except for ASD diagnosis, sex and age were first regressed out from the expression datasets. The co-expression networks and modules were estimated using the blockwiseModules function with the following parameters: corType=bicorr; networkType=signed; pamRespectsDendro=F; mergeCutHeight=0.1, power=8, deepSplit=2, minModuleSize=40. Module eigengene/genotype associations were calculated using a linear model. Significance p-values were FDR-corrected to account for multiple comparisons. Genes within each module were prioritized based on their module membership (kME), defined as correlation to the module eigengene. For selected modules, the top hub genes were shown.

Functional enrichment analysis

For co-expressed gene modules, enrichment for Gene Ontology (GO; Biological Process and Molecular Function) was performed using gProfileR R package (7). Background was restricted to

the expressed set of genes. An ordered query was used, ranking genes by kME for WGCNA analyses.

For DGE, GO enrichment was performed using the GSEA algorithm as implemented in the clusterProfiler R package (8). All genes were ranked by log2 fold change.

Enrichment analyses were also performed using several established, hypothesis-driven gene sets including pre- and postsynaptic marker genes (9), genes with likely-gene-disruption (LGD) or LGD plus missense de novo mutations(DNMs) found in patients with neurodevelopmental disorders (10), genes with probability of loss-of-function intolerance (pLI) > 0.99 as reported by the Exome Aggregation Consortium (11), mutationally constrained genes(12), vulnerable ASD genes(13), CHD8 targets(14), FMRP targets (15), syndromic and highly ranked (1 and 2) genes from SFARI Gene database. Statistical enrichment analyses were performed using permutation test. One thousand simulated lists with similar number of genes, gene length distribution and GC-content distribution as the target gene list were generated, and the overlaps between each of the simulated list and the hypothesis-driven gene sets were calculated to form the null distribution. Significance p-value was calculated by comparing the actual overlap between target list and hypothesis-driven gene sets to the null distribution. All results were FDR-corrected for multiple comparisons.

Cell type enrichment analysis

Cell-type enrichment analysis for each co-expression module was performed using the Expression Weighted Cell Type Enrichment (EWCE) package in R (16). Cell type-specific gene expression data was obtained from single nucleus sequencing of adult human brains (17). The specificity metric of each gene for each cell type was computed as described (16). Enrichment was evaluated using bootstrapping. Z-score was estimated by the distance of the mean

expression of the target gene set from the mean expression of bootstrapping replicates. P-values were corrected for multiple comparisons using FDR.

GWAS enrichment analysis

The most recent ASD GWAS summary statistics were obtained from Grove *et al.*(18) Stratified LD score regression (sLDSC) (19) was used to test whether a gene set of interest is enriched for SNP-heritability in a given GWAS dataset. In brief, SNPs were assigned to custom gene categories if they fell within ±100 kb of any gene in a set. These categories were added to a full baseline model that includes 53 functional categories capturing a broad set of genomic annotations. The MHC region was excluded from all analyses. Enrichment was calculated as the proportion of SNP-heritability accounted for by each category divided by the proportion of total SNPs within the category. Significance was assessed using a block jackknife procedure, followed by Bonferroni correction for the number of gene sets.

Ingenuity Pathway Analysis

We utilized Ingenuity Pathway Analysis (IPA®, QIAGEN) tools to predict the upstream regulators and to build mechanistic networks (20). The IPA's Mechanistic Network analysis allows for discovery of potential sets of interconnected upstream regulators that may drive the gene expression changes observed in ASD. In addition, IPA predicts the overall direction of the upstream regulator (activation or inhibition) from the IPA knowledge base using a Z-score (20). The following parameters were used in the mechanistic networks design: p of overlap for which upstream regulators to include was set to $p<0.05$; depth of the mechanistic network (# of steps between the regulators) = 2; breath of the mechanistic network (# of downstream regulators) = 5; filter out weaker relationships between regulators based on their overlapping targets: $p<0.005$; both direct and indirect interactions between regulators were considered.

Designing and learning the Bayesian network structure

Each of the 18 module eigengenes from our neuron co-expression data serves as one random variable (node) in our Bayesian network (BN). In addition, we also include diagnosis (ASD or CTL) as a binary random variable (node). This design allows us to distinguish between modules that are directly associated with ASD and those that are indirectly associated with ASD (21).

We discretized our eigengene expression data into three levels using Hartemink's method (22). We applied bootstrap resampling to the discretized data and learn a set of 500 network structures. One network structure was learned from each bootstrap sample using a hill-climbing search to optimize the BDe score (23). These 500 networks were then averaged to derive the consensus network, with arcs/edges considered significant if they appear in at least 67% of the networks and in the direction that appears most frequently.

Prioritizing genes within Neu-M5

The Bayesian network built from neuronal co-expression modules identified Neu-M5 as the module that is most strongly associated with ASD. To prioritize genes within the Neu-M5 module, we first identified Neu-M5 member genes that are also significant DEGs from our differential expression analysis, and this gave us 21 plausible genes. We then built Bayesian network among the 21 genes with method described in the above section. We selected genes with the highest number of connections as nodes likely to influence the expression of many other genes within the module.

Alzheimer's disease datasets

Alzheimer's disease differential gene expression data was obtained from the Mount Sinai/JJ Peters VA Medical Center Brain Bank (MSBB) study (24). RNA-seq data was available for 315 donors across 4 brain regions. To compare with our ASD results, we used the differential

expression data from STG. The MSBB differential expression data can be accessed through Synapse via accession number syn30821563.

SI References

1. Gandal MJ, Haney JR, Wamsley B, Yap CX, Parhami S, Emani PS, et al. Broad transcriptomic dysregulation occurs across the cerebral cortex in ASD. Nature. 2022:1-8.
2. Oldham MC, Langfelder P, Horvath S. Network methods for describing sample relationships in genomic datasets: application to Huntington's disease. BMC Syst Biol. 2012;6:63.
3. Love MI, Huber W, Anders S. Moderated estimation of fold change and dispersion for RNA-seq data with DESeq2. Genome biology. 2014;15(12):1-21.
4. Li YI, Knowles DA, Humphrey J, Barbeira AN, Dickinson SP, Im HK, et al. Annotation-free quantification of RNA splicing using LeafCutter. Nature genetics. 2018;50(1):151-8.
5. Gandal MJ, Zhang P, Hadjimichael E, Walker RL, Chen C, Liu S, et al. Transcriptome-wide isoform-level dysregulation in ASD, schizophrenia, and bipolar disorder. Science. 2018;362(6420).
6. Langfelder P, Horvath S. WGCNA: an R package for weighted correlation network analysis. BMC Bioinformatics. 2008;9:559.
7. Raudvere U, Kolberg L, Kuzmin I, Arak T, Adler P, Peterson H, et al. g: Profiler: a web server for functional enrichment analysis and conversions of gene lists (2019 update). Nucleic acids research. 2019;47(W1):W191-W8.
8. Yu G, Wang L-G, Han Y, He Q-Y. clusterProfiler: an R package for comparing biological themes among gene clusters. Omics: a journal of integrative biology. 2012;16(5):284-7.
9. Pirooznia M, Wang T, Avramopoulos D, Valle D, Thomas G, Huganir RL, et al. SynaptomeDB: an ontology-based knowledgebase for synaptic genes. Bioinformatics. 2012;28(6):897-9.
10. Turner TN, Yi Q, Krumm N, Huddleston J, Hoekzema K, F Stessman HA, et al. denovo-db: a compendium of human de novo variants. Nucleic Acids Res. 2017;45(D1):D804-D11.
11. Karczewski KJ, Weisburd B, Thomas B, Solomonson M, Ruderfer DM, Kavanagh D, et al. The ExAC browser: displaying reference data information from over 60 000 exomes. Nucleic Acids Res. 2017;45(D1):D840-D5.
12. Samocha KE, Robinson EB, Sanders SJ, Stevens C, Sabo A, McGrath LM, et al. A framework for the interpretation of de novo mutation in human disease. Nat Genet. 2014;46(9):944-50.
13. Iossifov I, Levy D, Allen J, Ye K, Ronemus M, Lee Y-H, et al. Low load for disruptive mutations in autism genes and their biased transmission. Proc Natl Acad Sci U S A. 2015;112(41):E5600-7.
14. Wilkinson B, Grepo N, Thompson BL, Kim J, Wang K, Evgrafov OV, et al. The autism-associated gene chromodomain helicase DNA-binding protein 8 (CHD8) regulates noncoding RNAs and autism-related genes. Transl Psychiatry. 2015;5:e568.

15. Darnell JC, Van Driesche SJ, Zhang C, Hung KYS, Mele A, Fraser CE, et al. FMRP stalls ribosomal translocation on mRNAs linked to synaptic function and autism. Cell. 2011;146(2):247-61.
16. Skene NG, Grant SG. Identification of vulnerable cell types in major brain disorders using single cell transcriptomes and expression weighted cell type enrichment. Frontiers in neuroscience. 2016;10:16.
17. Lake BB, Chen S, Sos BC, Fan J, Kaeser GE, Yung YC, et al. Integrative single-cell analysis of transcriptional and epigenetic states in the human adult brain. Nature biotechnology. 2018;36(1):70-80.
18. Grove J, Ripke S, Als TD, Mattheisen M, Walters RK, Won H, et al. Identification of common genetic risk variants for autism spectrum disorder. Nat Genet. 2019;51(3):431-44.
19. Finucane HK, Bulik-Sullivan B, Gusev A, Trynka G, Reshef Y, Loh P-R, et al. Partitioning heritability by functional annotation using genome-wide association summary statistics. Nature genetics. 2015;47(11):1228-35.
20. Krämer A, Green J, Pollard Jr J, Tugendreich S. Causal analysis approaches in ingenuity pathway analysis. Bioinformatics. 2014;30(4):523-30.
21. Mostafavi S, Gaiteri C, Sullivan SE, White CC, Tasaki S, Xu J, et al. A molecular network of the aging human brain provides insights into the pathology and cognitive decline of Alzheimer’s disease. Nature neuroscience. 2018;21(6):811-9.
22. Hartemink AJ. Principled computational methods for the validation discovery of genetic regulatory networks: Massachusetts Institute of Technology; 2001.
23. Heckerman D, Geiger D, Chickering DM. Learning Bayesian networks: The combination of knowledge and statistical data. Machine learning. 1995;20(3):197-243.
24. Wang M, Beckmann ND, Roussos P, Wang E, Zhou X, Wang Q, et al. The Mount Sinai cohort of large-scale genomic, transcriptomic and proteomic data in Alzheimer's disease. Scientific data. 2018;5(1):1-16.
